# Socioeconomic Status and Quality of Life: An Assessment of the Mediating Effect of Social Capital

**DOI:** 10.3390/healthcare11050749

**Published:** 2023-03-03

**Authors:** Jonathan Aseye Nutakor, Lulin Zhou, Ebenezer Larnyo, Stephen Addai-Danso, Debashree Tripura

**Affiliations:** 1 School of Management, Jiangsu University, Zhenjiang 212013, China; 2 Center for Black Studies Research, University of California, Santa Barbara, CA 93106, USA

**Keywords:** socioeconomic status, social capital, quality of life, EUROHIS-QOL

## Abstract

Socioeconomic status has been found to be a significant predictor of quality of life, with individuals of higher socioeconomic status reporting better quality of life. However, social capital may play a mediating role in this relationship. This study highlights the need for further research on the role of social capital in the relationship between socioeconomic status and quality of life, and the potential implications for policies aimed at reducing health and social inequalities. The study used a cross-sectional design with 1792 adults 18 and older from Wave 2 of the Study of Global AGEing and Adult Health. We employed a mediation analysis to investigate the relationship between socioeconomic status, social capital, and quality of life. The results showed that socioeconomic status was a strong predictor of social capital and quality of life. In addition to this, there was a positive correlation between social capital and quality of life. We found social capital to be a significant mechanism by which adults’ socioeconomic status influences their quality of life. It is crucial to invest in social infrastructure, encourage social cohesiveness, and decrease social inequities due to the significance of social capital in the connection between socioeconomic status and quality of life. To improve quality of life, policymakers and practitioners might concentrate on creating and fostering social networks and connections in communities, encouraging social capital among people, and ensuring fair access to resources and opportunities.

## 1. Introduction

Extensive research has shed light on the relationship between one’s socioeconomic status and their level of health and overall quality of life [1]. In addition, research conducted over the course of the past two decades has uncovered a correlation between social capital and quality of life [2,3]. In recent years, efforts to identify and address the social determinants of people’s health and quality of life have led to widespread acknowledgment of certain elements of social capital as complements to public health measures [4,5].

Social capital remains a highly effective idea that has been thought about from a range of viewpoints, yet a number of its qualities have been utilized in public health research. The ability of actors, be they individuals or groups, to obtain benefits by virtue of their participation in social networks and other social structures is one definition of social capital [2]. Whereas Coleman, Bourdieu, and Putnam defined social capital from different viewpoints [6,7,8], they all acknowledged it as a significant social asset and as individual and group characteristics that can be quantified and assessed within a social network for health and wellbeing advantages. This study defines social capital as a useful social resource that adults can use at the individual and community levels to improve their health and quality of life. These resources can come from families, schools, colleagues, and community members. They can be acquired to get the most health benefits and possibly protect the quality of life of adults from the impacts of socioeconomic inequality [9,10,11]. Some individuals have the ability to leverage the pre-existing resources of their family or their participation in influential groups or associations to their benefit. Social capital is a collective asset that is advantageous to everyone who is a part of a social structure [12]. It is an asset not only for wealthy and privileged people, but also for those who are less privileged [13]. Social capital is built, maintained, and sustained with the help of trust, cooperation, and reciprocity [14]. Social capital is a health determinant because social ties and interactions may provide information and real support. The way in which a society’s social structure, as well as its ideas of familial relationships and social connections, develops throughout the course of time is directly related to the way in which social capital changes over time [15,16]. As a result, certain efforts have been made to build social capital because it can be beneficial in improving people’s health, presumably regardless of their wealth status [13].

The concept of social capital appears to incorporate aspects of both psychosocial factors and social wellbeing. This is due to the fact that it serves as an essential channel through which an adult’s social environment can influence the majority of the factors that comprise their quality of life [9,17]. However, social inequality in adults’ quality of life is frequently quantified by looking at differences in adults’ financial status, while social capital is largely ignored as a measurement tool. This is despite the fact that social capital is one of the most important factors in adulthood. It has been discovered that one’s socioeconomic situation has an effect on their health and quality of life both directly [9,10,17,18,19] and indirectly through psychosocial factors such as the failure of the poor to form ties and networks for their own advantage [8,9,17]. However, these psychosocial characteristics have the potential to operate as protective mechanisms, which can help mitigate some of the negative consequences that socioeconomic status disparities have on quality of life. This is due to the fact that research has shown that a person’s socioemotional and psychosocial resources, which they build up via their relationships with others in their social environment, can attenuate the negative impacts of their socioeconomic status on their quality of life [9,10,17]. This supports the idea that social capital may serve as a buffer against health problems, particularly for those from economically disadvantaged backgrounds.

In addition, social capital is widely recognized as a key determinant of quality of life because it can provide individuals with important support and resources that enhance their well-being. Quality of life is an essential indicator of human health that is influenced by physical, mental, and social factors [20]. Quality of life has to do with how satisfied or happy a person is, which is affected by health. It is a key indicator of both physical and mental health. Negative social capital, on the other hand, can have detrimental consequences on the relationship between socioeconomic status and quality of life, especially in the setting of in-group violence and gang activities. Negative social capital can contribute to the propagation of negative stereotypes and stigmatization of specific groups, resulting in their further marginalization and exclusion from society. In addition, it can destroy social trust, resulting in feelings of isolation and separation.

Recognizing the protective roles of social capital can help address the differences in quality of life between adults based on their socioeconomic status. This is especially important in low–and middle–income countries, where many adults face risks related to poverty and low socioeconomic status. On the other hand, there is a dearth of empirical evidence about the protective function of social capital using a nationally representative sample of adults in Ghana and most low-and middle-income economies. As a result, the purpose of this study is to investigate the potential for social capital to mediate the relationship between an adult’s socioeconomic status and their quality of life using nationally representative data from a low-and middle-income country [Ghana]. In light of the foregoing, we employed a mediation analysis to examine the interplay between socioeconomic status, social capital, and quality of life.

## 2. Materials and Methods

This study utilized data from SAGE Wave 2 of the World Health Organization’s Study on Global AGEing and Adult Health. SAGE was carried out in six low- and middle-income countries (China, Ghana, India, Mexico, Russian Federation, and South Africa). There are 4 waves in WHO SAGE, 0, 1, 2 and 3. This study focused on wave 2 because wave 3 is yet to be made available to the public. The data collected in Ghana was utilized for this study. This study’s total sample size was 1792 respondents. This group consisted of adults older than 18 years. In order to accommodate respondents in Ghana who lacked English language skills, the questionnaire used for this research was translated into a variety of the country’s native tongues. The WHO Ethical Review Committee (RPC146) and the University of Ghana Medical School Ethics and Protocol Review Committee approved SAGE [Accra, Ghana] [21].

### 2.1. Measures

#### 2.1.1. Socioeconomic Status

Socioeconomic status was defined using a combination of education and household income. Five groups were made based on the level of education of the respondents. These included individuals who have completed less than primary school, primary school, secondary school, high school, and a university/college/postgraduate degree [22]. Likewise, household income was categorized into five groups. These ranged from the lowest to the highest income brackets [23]. We combined the two variables to create an overall socioeconomic status score. The total socioeconomic status score ranged from 1 to 5, with higher scores indicating higher socioeconomic status.

#### 2.1.2. Quality of Life

The EUROHIS-QOL 8-item index was utilized in order to do the analysis for quality of life [24]; this index is the condensed version of the Quality-of-Life Instrument that was developed by the World Health Organization. In addition, the EUROHIS-QOL 8-item measure has been shown to have validity and reliability that are satisfactory [25]. The questions used to assess quality of life were, (1) Do you have enough energy for everyday life?, (2) Do you have enough money to meet your needs?, (3) How satisfied are you with your health?, (4) How satisfied are you with yourself?, (5) How satisfied are you with your ability to perform your daily living activities?, (6) How satisfied are you with your personal relationships?, (7) How satisfied are you with the conditions of your living place?, and (8) How would you rate your overall quality of life? The total score for these eight questions was between 8 and 40, where 8 was the lowest possible score, and 40 was the highest. Respondents who were unsatisfied with all areas of their quality of life received the lowest possible score, while those who were extremely satisfied with all aspects of their wellbeing received the highest possible score.

#### 2.1.3. Social Capital

In order to assess social capital, two different variables were constructed. These included social participation and trust. The determinants of social capital were derived from a combination of fourteen separate questions. In order to determine whether or not the respondents had a trusting relationship with the individuals [friends, neighbors, and family] in their immediate environment, questions pertaining to trust were posed to them. In addition, questions on social participation focused on the participants’ involvement with family, friends, neighbors, and public gatherings. Assessing social capital is a key method for assessing the extent of social cohesiveness and collective activity in a society. Trust and social participation are important indicators of social capital in terms of its role as a mediator between socioeconomic status and quality of life [26]. Social capital may promote the wellbeing of individuals through enhancing social interaction, resource access, and resource utilization. The operationalization of social capital does not follow a one-size-fits-all model, and various studies may utilize different measures based on their study objective and environment. Furthermore, social capital may be measured at several levels, including the individual, community, and societal levels.

#### 2.1.4. Sociodemographic

There were male and female codes for gender. Age was coded and classified into seven distinct groups. These age groups ranged from 18 to 24, 25 to 34, 35 to 44, 45 to 54, 55 to 64, 65 to 74, and 75 and older. The categories of urban and rural were assigned based on the resident’s location. There were five distinct categories of people based on their marital status. They included those who have never been married, those who are currently married, those who cohabit, those who are separated or divorced, and those who have lost a spouse. 

### 2.2. Statistical Analysis

Analyses of the data were performed using STATA SE 14.2 (Stata Corp., College Station, TX, USA) and Intellectus Statistics. The following categories of summary statistics were calculated: gender, age, residence, marital status, education level, and income quintile. A causal mediation study was carried out to determine if social capital mediated the relationship between socioeconomic status and quality of life. The mediation model was based on an exposure variable, mediator, and an outcome variable. In order to test the normality assumption, the quantiles of the model residuals were plotted against the quantiles of a Chi-square distribution. This allowed for an accurate evaluation of the assumption. The homoscedasticity of the data was determined by making a scatter plot of the residuals vs. the anticipated values. The occurrence of multicollinearity between predictors was investigated through the use of variance inflation factors (VIFs), which were computed. When *p* was less than 0.05, the results of the study were regarded as significant.

## 3. Results

The total sample size for this study was 1792 respondents. It was found that females made up 50.33 percent of the total respondents. The group of adults aged 55 to 64 made up the largest portion of the sample (26.45%). When it came to the respondents’ places of residence, those who lived in rural areas made up the majority of the group (51.95%). The marital status of the respondents was assessed as part of the study. According to the data, the group of respondents who are currently married made up 57.09 percent of the total. In terms of the respondents’ educational backgrounds, the largest group was comprised of those who had completed secondary school (26.34%). Finally, we divided the respondents’ household income into five categories and evaluated the results. According to the findings of our survey, the majority of respondents, or 38.62 percent, were positioned in the lowest quintile. The summary statistics can be found in Table 1.

**Figure 1:**
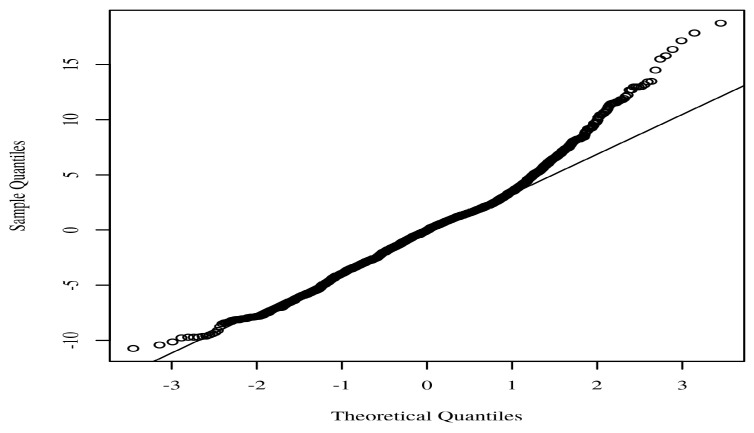
Regression model Q-Q scatterplot for residual normality.

**Figure 2:**
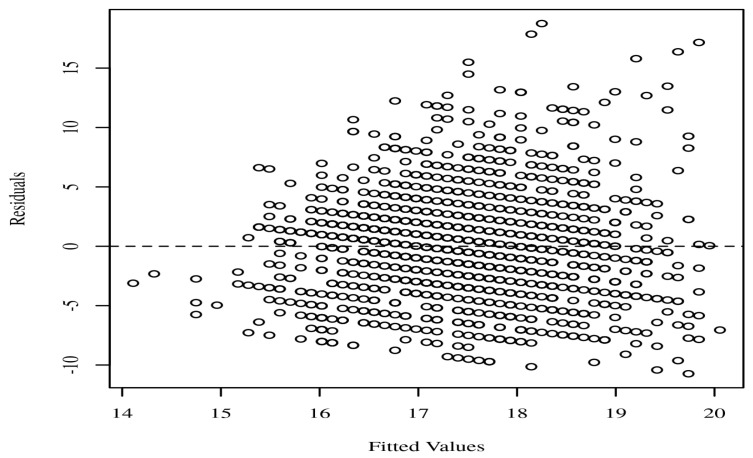
Residuals scatterplot testing homoscedasticity.

It is essential that the quantiles of the residuals do not significantly depart from the theoretical quantiles for the assumption of normality to be validated. The reliability of the parameter estimates may be called into question if there is a significant deviation. A Q-Q scatterplot of the model residuals is shown in Figure 1. If the points seem to be randomly distributed, have a mean of zero, and there is no visible curvature, then the assumption of homoscedasticity has been satisfied. A scatterplot of the anticipated values and the model residuals can be seen in Figure 2. In the regression model, each of the predictors has a VIF that is less than 10. The VIF for each predictor included in the model is detailed in Table 2.

**Figure 3:**
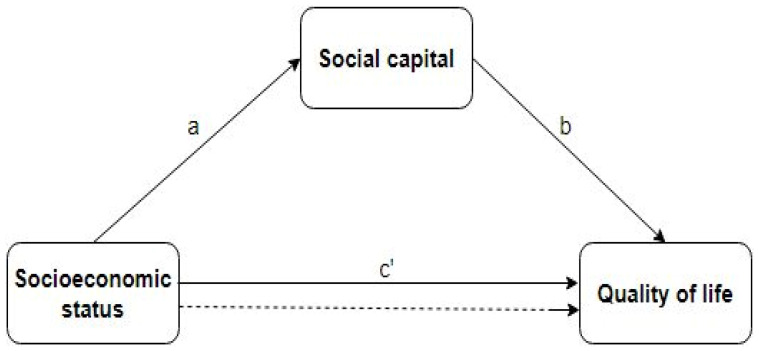
Node diagram for the mediation analysis. The dotted line represents the effect of socioeconomic status on quality of life, when social capital is not taken into account as a mediator. The paths a, b and c’ are direct effects.

Using bootstrapping (N = 100) and confidence intervals based on percentiles, the mediating effects were investigated based on the indirect and direct effects. The outcomes were determined using an alpha of 0.05. Figure 3 displays a diagram of the mediation model.

As shown in Table 3, there is a positive relationship between socioeconomic status and quality of life. The direct effect on average was significant, with a value of *B* = 0.21, 95% CI [0.14, 0.34], *p* < 0.021. This shows that socioeconomic status is a strong predictor of life quality. In Table 4, a similar association was found between social capital and quality of life. Thus, the direct effect of social capital on quality of life was significant, with a value of *B* = 1.49, 95% CI [1.11, 1.56], *p* < 0.001. In Table 5, a positive relationship was observed between socioeconomic status and social capital *B* = 0.06, 95% CI [0.03, 0.08]. Through social capital, the average indirect effect of socioeconomic status on quality of life was significant (*B* = 0.09, 95% CI [0.03, 0.15]) (Table 6). In other words, the effect of socioeconomic status on quality of life was mediated by social capital.

## 4. Discussion

Consistent with the World Health Organization’s Social Determinants of Health framework, we discovered evidence that social capital mediates the effect of socioeconomic status on quality of life [27]. This finding is also consistent with a systematic review that was conducted in 2013 and came to the conclusion that social capital might be able to buffer the adverse impacts of poor socioeconomic status on one’s health [28]. The World Health Organization’s Social Determinants of Health framework recognizes that social factors such as social capital, socioeconomic status, and other social determinants, have a significant impact on health outcomes and quality of life [29]. According to a research conducted in Canada, social capital mediates the relationship between education and self-rated health [30]. In the United Kingdom, a study showed that social capital mediated the relationship between income and self-rated health [31]. Other research showed opposite findings to our own. In the United States, a study showed that social capital did not mediate the association between socioeconomic status and self-rated health [32]. Also, a similar study showed that social capital did not mediate the association between socioeconomic status and health-related quality of life [33]. Overall, these findings imply that social capital may not always serve as a mediator between socioeconomic status and quality of life and that other factors may be at play. The relationship between socioeconomic status and quality of life is complex and multifaceted.

The mediation of the influence of socioeconomic status on quality of life by social capital in this setting shows that social capital plays a crucial role in the relationship between socioeconomic status and quality of life. This study shows that persons with greater levels of social capital are better equipped to harness the benefits associated with a higher socioeconomic status in order to obtain a higher quality of life. Social capital may give access to resources and opportunities that are unavailable to others without such connections, and it can contribute to the development of an essential sense of social support and belonging [12]. In addition, the fact that social capital mediates the relationship between socioeconomic status and quality of life shows that social capital might mitigate the detrimental impacts of social and economic disadvantage on health and well-being [28]. This is a significant finding, since it shows that interventions focused at developing and sustaining social capital may have the potential to enhance health outcomes and minimize health inequalities across people and groups. Overall, the findings that social capital mediates the influence of socioeconomic status on quality of life supports a growing corpus of research on social determinants of health and emphasizes the significance of strong social ties and relationships to increase well-being [28]. The results of this study also show that health systems need to do more than just provide preventive and curative care. They also need to put money into programs that help people with low incomes build or keep social capital. These programs may include community development programs, mentoring programs, skill-building programs, and support groups. These programs focus on building community connections, providing opportunities for skill-building and support, and creating a sense of belonging and empowerment.

According to our study, socioeconomic status and quality of life are positively correlated. Another study with similar results supports this [34]. Research have repeatedly demonstrated that socioeconomic status has a strong direct impact on quality of life [5,13,34,35,36]. Comparatively to individuals with lower socioeconomic status, those with higher socioeconomic status typically enjoy higher quality of life [37]. This is because having a higher socioeconomic standing frequently translates into having access to better healthcare, employment prospects, and educational institutions, as well as more income and wealth [38]. More stable living conditions, such as having access to a clean and safe place to live, reliable transportation, and other basic necessities are also related to higher socioeconomic status [39]. All of these elements may lead to improved physical and mental health outcomes, more sociable engagement and community involvement, and higher levels of personal contentment. Although socioeconomic status may have a big influence on quality of life, the relationship between the two is complex and multifaceted. An individual’s opinion of their quality of life may also be influenced by other elements including personal values, cultural background, and life events.

In a similar vein, a positive correlation was found to exist between a person’s socioeconomic status and their social capital. A positive association between socioeconomic status and social capital indicates that when a person’s socioeconomic standing rises, so does their social capital. Higher social capital has been shown to correlate positively with higher socioeconomic status in recent research [5,13,40]. This association makes logical sense, since individuals with a better socioeconomic standing often have more resources and chances to establish and maintain social connections. They may have access to better schools, employment opportunities, and other social and cultural organizations, which can facilitate the formation and maintenance of important social bonds [41]. In exchange, these social relationships can give the individual extra resources and possibilities, such as information access, employment leads, and other types of assistance. Overall, the positive correlation between socioeconomic status and social capital suggests that individuals with higher socioeconomic status are more likely to have a broader and more diverse network of social connections, which can provide them with significant advantages in a variety of aspects of their lives. Again, we found a positive relationship between social capital and quality of life, which is consistent with previous research that found a lack of social contact is strongly associated with low quality of life [40]. A positive correlation between social capital and quality of life indicates that as social capital improves, so does people’ quality of life. This association makes sense since social capital may provide individuals with a variety of resources and possibilities, including social support, access to knowledge and resources, and social and economic development prospects [28]. These resources can contribute to a greater quality of life by enhancing health, fostering social connections and a feeling of belonging, and offering access to resources and opportunities that may not be available to persons without these connections. In addition, social capital can facilitate the development of trust and collaboration between individuals, resulting in more stable and resilient societies [42].

This study adds to the existing literature by showing that social capital mediates the relationship between socioeconomic status and quality of life [9,10,43,44]; yet, it is still an original finding with significant implications. Despite the fact that earlier studies have shown that both socioeconomic status and social capital are important in determining health outcomes, this study provides evidence that social capital plays a crucial role in relating socioeconomic status to quality of life. In other words, it emphasizes the importance of interpersonal interactions in achieving the benefits associated with a greater socioeconomic status. This conclusion is especially significant because it shows that initiatives designed to improve health outcomes should prioritize the development and maintenance of networks of social support rather than merely increasing access to financial resources or encouraging healthier individual behaviors. This is a departure from the standard public health practice, which has concentrated on treatments at the individual level, and an advancement toward one that takes into account the impact of social determinants of health. In addition, the focus of this study on social capital as a mediator of the association between socioeconomic status and quality of life is a unique contribution to the field. Policymakers and practitioners may need to concentrate on lowering inequalities in social capital to reduce inequalities in quality of life. Efforts to improve educational and economic prospects for those with a low socioeconomic status, for instance, may raise their social capital and, in turn, their quality of life.

The findings of this study have implications for future research which can also be utilized in the formation of national policies and programs that are geared toward the general population. More study is required to understand the mechanisms through which social capital functions as a mediator, as well as the variables that impact the development and distribution of social capital. In addition, research might investigate the efficacy of certain treatments targeted to improve social capital among persons of low socioeconomic status. Health professionals and policymakers both have a responsibility to see that older adults receive the best possible care, both for their physical and functional needs and for their emotional and psychological well-being. This includes helping them feel more useful, which has been linked to better physical functioning [45], by getting them more involved in their communities and letting them help make decisions at home.

Several theoretical approaches can be drawn from the finding that social capital influences the relationship between socioeconomic status and quality of life. First, it supports the social capital theory, which suggests that social connections, networks, and norms are valuable resources that can help people and communities do better [46]. The study implies that social capital can provide those with lower socioeconomic status with access to resources and assistance that enhance their quality of life. Second, the study emphasizes the significance of social factors in understanding health inequalities. It shows that treatments that promote social capital may be an effective means of reducing disparities in the quality of life between people of various socioeconomic backgrounds. Lastly, the study emphasizes the importance of policies that promote the growth of social capital. For instance, policies that stimulate social involvement, promote community development, and support the establishment of social networks may be crucial for enhancing the quality of life for those with a lower socioeconomic status.

The strength of this study lies in the fact that it is one of the few that analyze the relationship between socioeconomic status, social capital, and quality of life using a nationally representative sample of adults from a low- and middle-income country. There are some limitations to this study. Because this was a cross-sectional study, we are unable to determine whether socioeconomic status or social capital has a causal effect on quality of life. However, the proposed mediation model is in line with the WHO’s Social Determinants of Health Framework, providing evidence for the potential causal impacts of socioeconomic status and social capital on quality of life. Cross-sectional studies are not designed to provide causal inferences because all data are collected at a single point in time. On the basis of the results of a cross-sectional research study alone, it is not possible to tell whether socioeconomic status or social capital has a causal influence on quality of life. The Social Determinants of Health framework acknowledges that social and economic factors, such as socioeconomic status and social capital, may have a substantial influence on an individual’s health and well-being. To prove causality, future research would require longitudinal or experimental designs to determine their impacts on quality of life over time. Nonetheless, the current study provides a solid foundation for future research in this area and underlines the potential significance of social capital in enhancing health and well-being. In addition, the majority of the responses to the questions were self-reported, which means that there is a possibility of recollection bias and that the results encounter reliability issues. Finally, the sample’s sociodemographic and socioeconomic characteristics are very varied. This may be observed in the size disparity between individuals who attended university and those who did not. It’s possible that the socioeconomic status of the university’s subjects is higher than that of the non-graduates.

## 5. Conclusions

The findings of this study have substantial implications for public health policy and practice because they imply that interventions focused at boosting social capital might assist to minimize health inequalities and improve health outcomes, especially among disadvantaged groups. The findings of the study underscore the need for more research into the particular processes through which social capital improves health outcomes, as well as the long-term benefits of social capital interventions on health outcomes. It is crucial to emphasize that this is a cross-sectional research study, therefore it cannot show a causal relationship. Yet, the results of this study provide a solid framework for future research and emphasize the potential significance of social capital in enhancing health and well-being.

## Figures and Tables

**Table 1:** Summary Statistics Table for Interval and Ratio Variables.

Variable	*Frequency* *n = 1792*	*%*
Gender		
Male	890	49.67
Female	902	50.33
Age		
18–24	136	7.59
25–34	195	10.88
35–44	236	13.17
45–54	314	17.52
55–64	474	26.45
65–74	288	16.07
75+	149	8.31
Residence		
Urban	861	48.05
Rural	931	51.95
Marital status		
Never married	234	13.06
Currently married	1023	57.09
Cohabiting	43	2.4
Separated/Divorced	241	13.45
Widowed	251	14.01
Education		
Less than primary school	426	23.77
Primary school completed	442	24.67
Secondary school completed	472	26.34
High school completed	357	19.92
University/Post graduate degree completed	95	5.3
Income quintile		
Lowest	692	38.62
2	155	8.65
3	267	14.9
4	373	20.81
Highest	305	17.02

*n* = Sample size; % = Percent.

**Table 2:** Variance Inflation Factors for Socioeconomic status and social capital.

Variable	VIF
Socioeconomic status	1.01
Social capital	1.01

VIF—Variance Inflation Factor.

**Table 3:** Direct effect of socioeconomic status on Quality of life.

				95% Confidence Interval	
	*B*	*SE*	*t*	Lower	Upper
Socioeconomic status → Quality of life	0.21 *	0.09	2.3	0.14	0.34

* *p* < 0.05; *B*—Unstandardized Beta; *SE*—Standard Error; CI—Confidence Interval; *t*—*t*-Test Statistic.

**Table 4:** Direct effect of social capital on Quality of life.

				95% Confidence Interval	
	*B*	*SE*	M	Lower	Upper
Social capital → Quality of life	1.49 ***	0.17	8.89	1.11	1.56

*** *p* < 0.001; *B*—Unstandardized Beta; *SE*—Standard Error; CI—Confidence Interval; M—Mean.

**Table 5:** Direct effect of socioeconomic status on Social capital.

				95% Confidence Interval	
	*B*	*SE*	*t*	Lower	Upper
Socioeconomic status → Social capital	0.06 ***	0.01	4.55	0.03	0.08

*** *p* < 0.001; *B*—Unstandardized Beta; *SE*—Standard Error; CI—Confidence Interval; *t*—*t*-Test Statistic.

**Table 6:** Indirect effect of Socioeconomic status on Quality of life through Social capital.

				95% Confidence Interval	
	*B*	*SE*	M	Lower	Upper
Socioeconomic status → Social capital → Quality of life	0.09 ***	0.02	3.27	0.03	0.15

*** *p* < 0.001; *B*—Unstandardized Beta; *SE*—Standard Error; CI—Confidence Interval; M—Mean.

## Data Availability

Publicly available datasets were analyzed in this study. This data can be found here: https://www.datafirst.uct.ac.za/dataportal/index.php/catalog/NIDS-CRAM (accessed on 6 December 2021).
